# Supplementary material for: Mechanisms of redundancy and specificity of the Aspergillus fumigatus Crh transglycosylases
Source: Nat Commun. 2019 Apr 10;10:1669. doi: 10.1038/s41467-019-09674-0 (PMC6458159; doi:10.1038/s41467-019-09674-0)
Supplement: Supplementary file 1 — Supplementary Information [file 41467_2019_9674_MOESM1_ESM.pdf]

Supplementary Information

Mechanisms of redundancy and specificity of the *Aspergillus fumigatus* Crh transglycosylases

Fang et al.

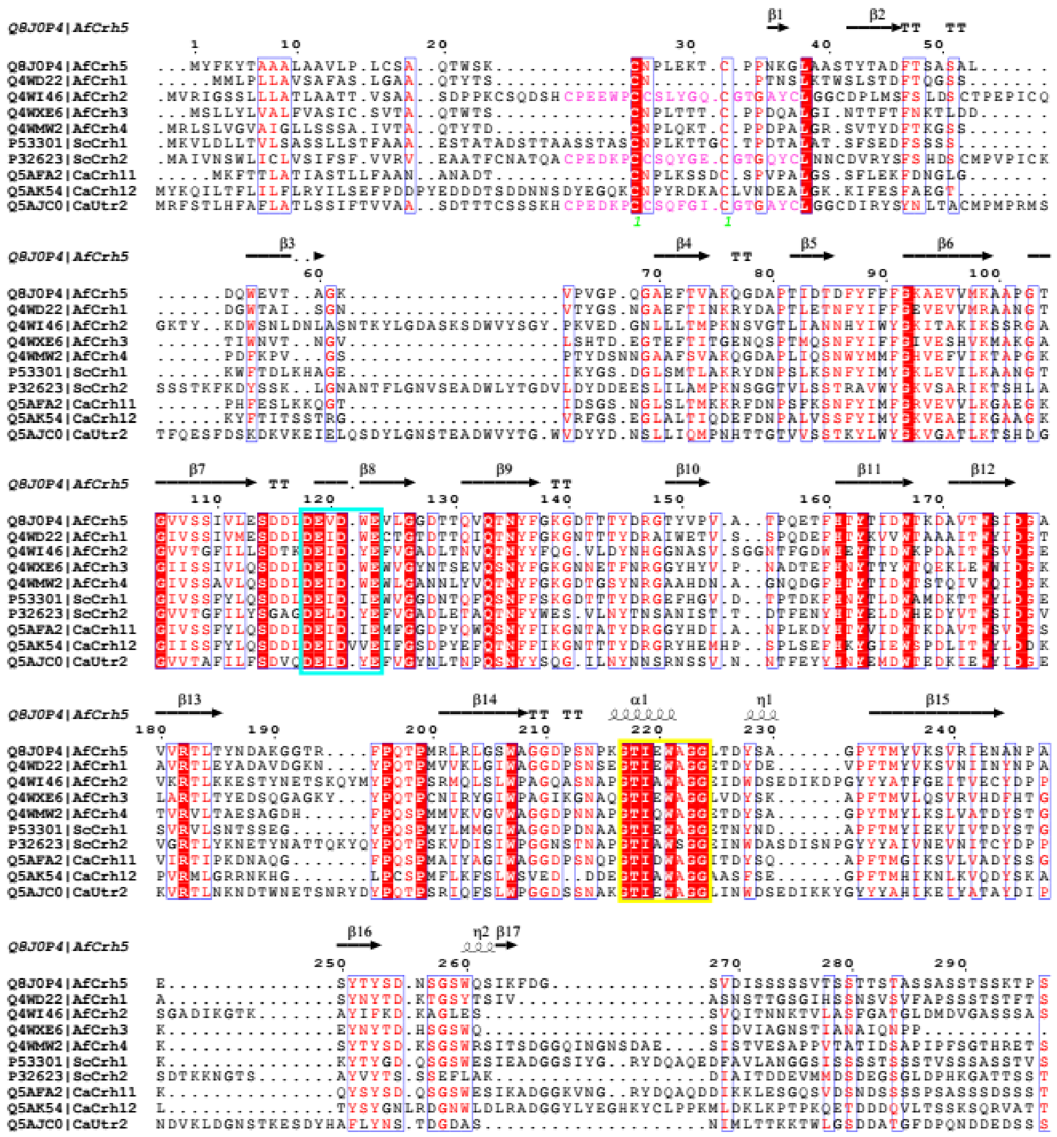

Supplementary Figure 1. Sequence alignment of *A. fumigatus* Crh enzymes with orthologues from *S. cerevisiae* and *C. albicans*. Due to the low sequence identity at the *C*-terminus residues 1-297 of *Af*Crh5 were used for alignment, which was performed by ClustalW. The secondary structural elements (helix-α, strand-β, 3_{10} helix-η, turn-TT) of *Af*Crh5 are shown above the sequences. The conserved catalytic motif is boxed by a cyan rectangle; the acceptor sugar binding motif is boxed by a yellow rectangle. The *N*-terminal CBM module is highlighted as magenta. The two cysteines indicated by light green markers below the sequence form a disulfide bridge. The figure was produced by ESPript1 [1].

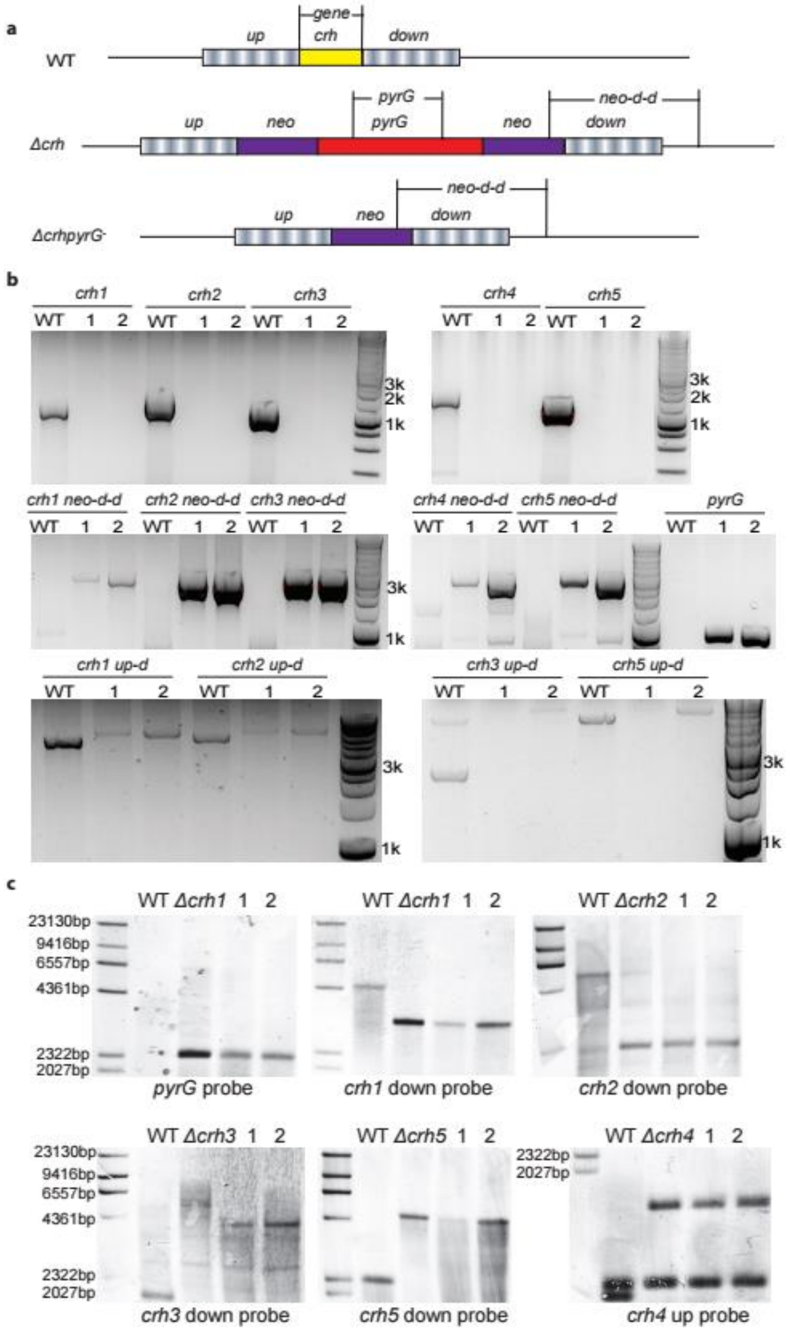

Supplementary Figure 2. Generation and confirmation of *crh* mutants. a) Schematic diagram of reusing the selective marker *pyrG*. b) PCR confirmation of the crh mutants using primer pairs of P31 & P32, P33 & P34, P35 & P36, P37 & P38 and P39& P40 to amplify *crh1*, *crh2*, *crh3*, *crh4* and *crh5* genes, respectively. Primers P23 & P24 were used to amplify *pyrG* gene; primer P25 paired with P26, P27, P28, P29 and P30 to amplify *crh1 neo-d-d*, *crh2 neo-d-d*, *crh3 neo-d-d*, *crh4 neo-d-d* and *crh5 neo-d-d* fragments; primer pairs of P3 & P6, P7 & P10, P11 & P14 and P19 & P22 to amplify upstream-gene-downstream parts of crh1, crh2, crh3 and crh5, respectively. c)

Southern blot confirmation using *pyrG*, *crh1* downstream, *crh2* downstream, *crh3* downstream, *crh4* upstream and *crh5* downstream as probes. Strain 1 and 2 represent quintuple mutant candidates. Source data are provided as a Source Data file.

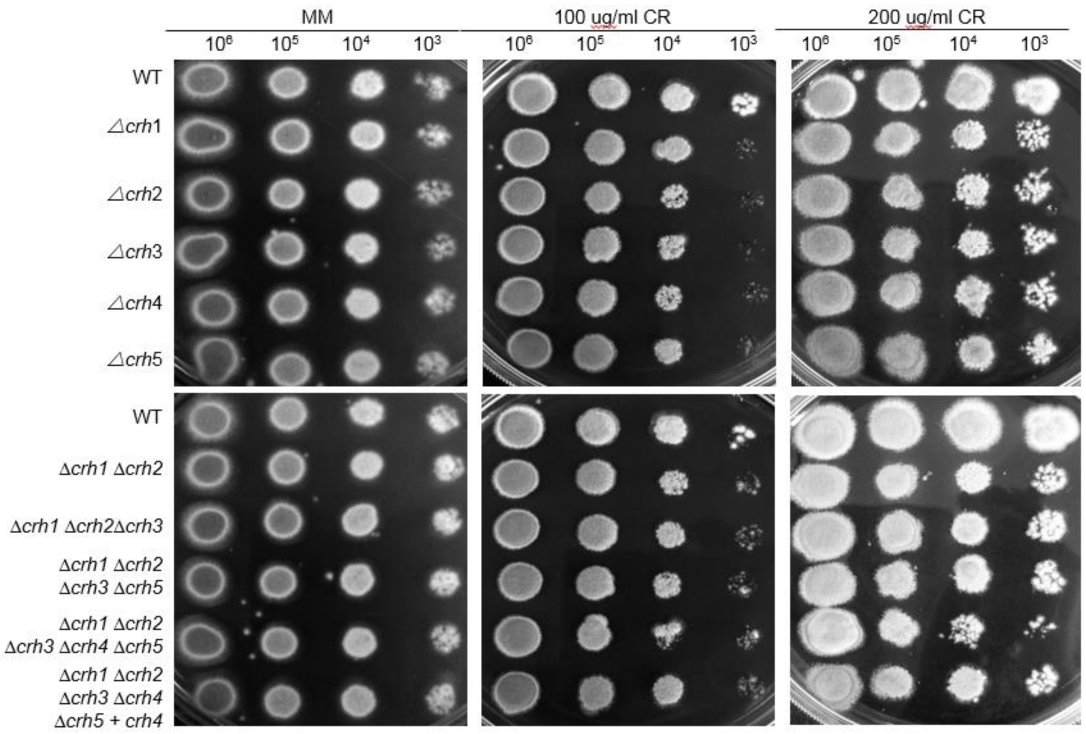

Supplementary Figure 3. Deletion of *crh* genes renders slight sensitivity to Congo red. The indicated strains were spotted on MM plates in the absence or presence of 100 µg ml^{-1} and 200 µg ml^{-1} of CR. Photos were taken after incubation at 37 °C for 48 h for MM and 100 µg ml^{-1} CR plates or 72 h for 200 µg ml^{-1} CR plate. Source data are provided as a Source Data file.

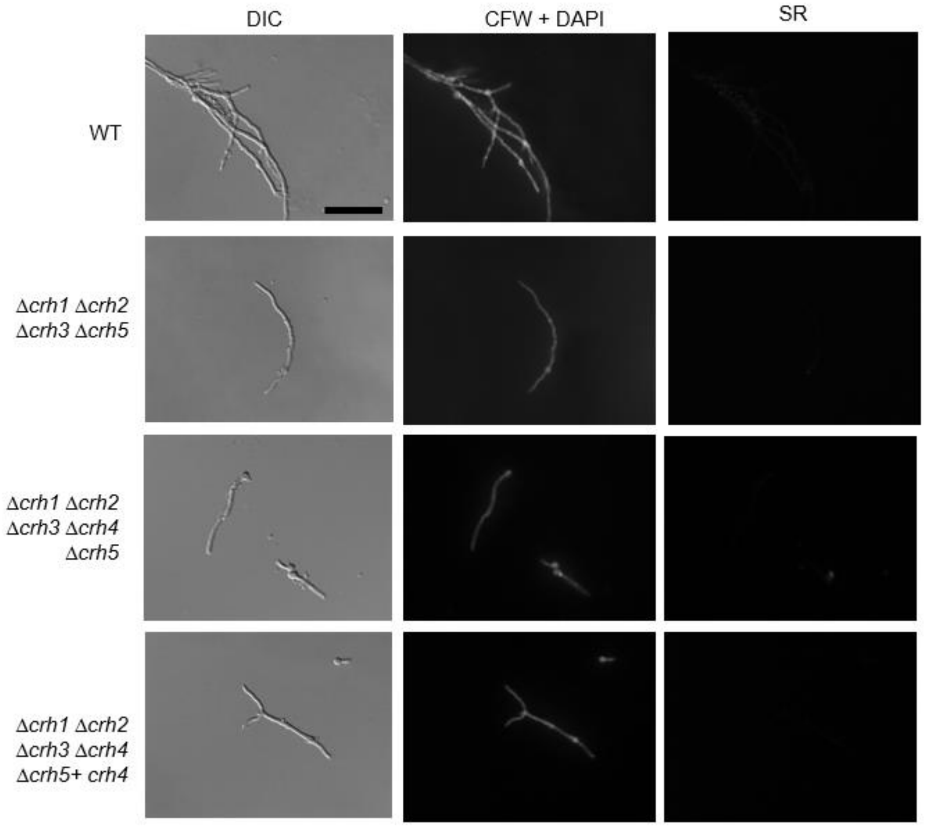

Supplementary Figure 4. Incorporation of sulphorhodamine (SR) in *A. fumigatus*. 10^5 conidia of WT and indicated strains were incubated with 3.75 µM of SR in MM for 16 h at 37 °C. Cells were fixed and stained with 10 µg ml^{-1} DAPI and 10 µg ml^{-1} CFW before being analysed by fluorescent microscopy. All images were taken at the same exposure. Scale bar, 50 µm.

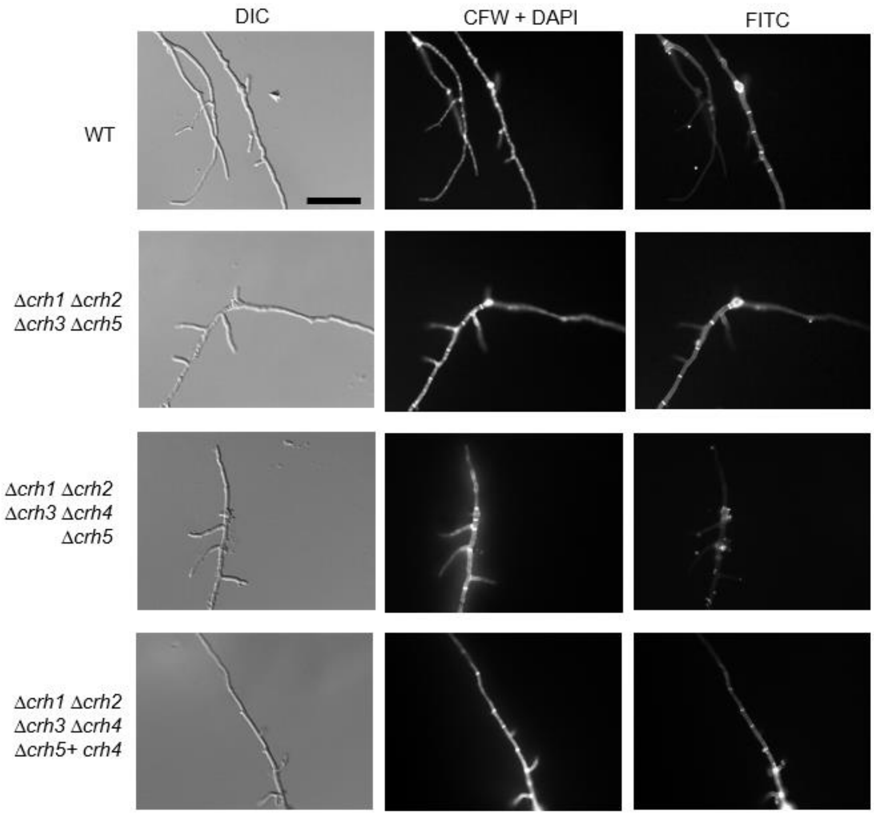

Supplementary Figure 5. Incorporation of FITC labelled NAG6 (NAG6-FITC) in *A. fumigatus*. 10^5 conidia of WT and indicated strains were incubated with 3.75 µM of NAG6-FITC in MM for 16 h at 37 °C. Cells were fixed and stained with 10 µg ml^{-1} DAPI and 10 µg ml^{-1} CFW before being analysed by fluorescent microscopy. All images were taken at the same exposure. Scale bar, 50 µm.

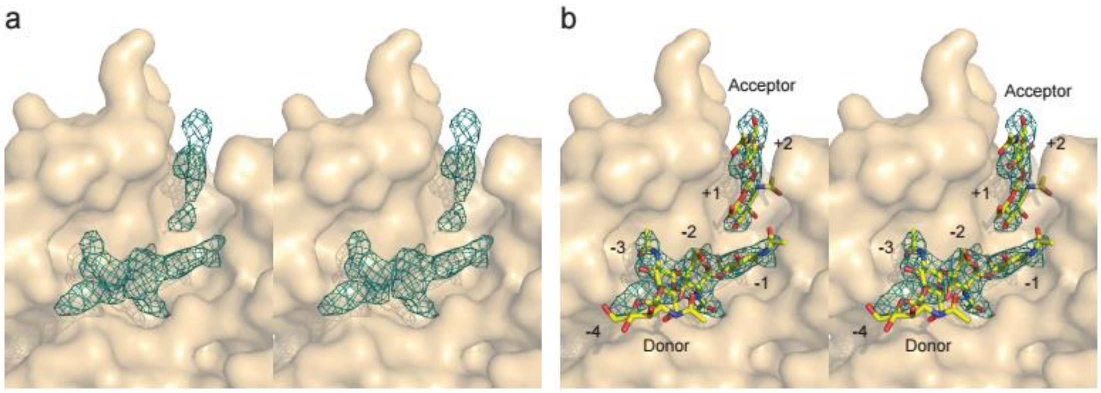

c

Atom type	Number	Average B	Maximum B	Average Q
Overall				
Protein main chain	1936	28.027	85.885	1.000
Protein side chain	1738	29.847	77.175	1.000
Protein all atoms	3674	28.888	85.885	1.000
Ligand all atoms	172	51.281	83.125	1.000
Water molecules	46	14.235	29.760	1.000
All atoms	3892	29.705	85.885	1.000
Full Ocupancy				
Donor (-1 to -4)	57	45.837	73.410	1.000
Donor (-1, -2)	29	39.535	53.290	1.000
Donor (-3)	14	39.935	52.535	1.000
Donor (-4)	14	64.792	73.410	1.000
Acceptor (+1,+2)	29	61.980	83.125	1.000
Adjusted Ocupancy				
Donor (-1 to -4)	57	31.201	48.770	0.800
Donor (-1, -2)	29	32.134	44.760	0.900
Donor (-3)	14	24.638	27.640	0.800
Donor (-4)	14	27.958	29.235	0.600
Acceptor (+1,+2)	29	27.420	34.665	0.600

Supplementary Figure 6. Stereo view of the ligand omit map. a) The blue mesh cage represents a 2 σ countored electron density from 20-fold average omit map wherein the NAG molecules were excluded from the initial phase calculation and further map averaging. b) The NAG4 molecules, represented as yellow sticks, as fitted into the omit map. c) Table showing the overall calculated B values at 100% occupancy (Q) plus the extracted values for both donor and acceptor and the further manually adjusted Q values to match the protein observed B values. Calculations were performed with Moleman (USF-uppsala).

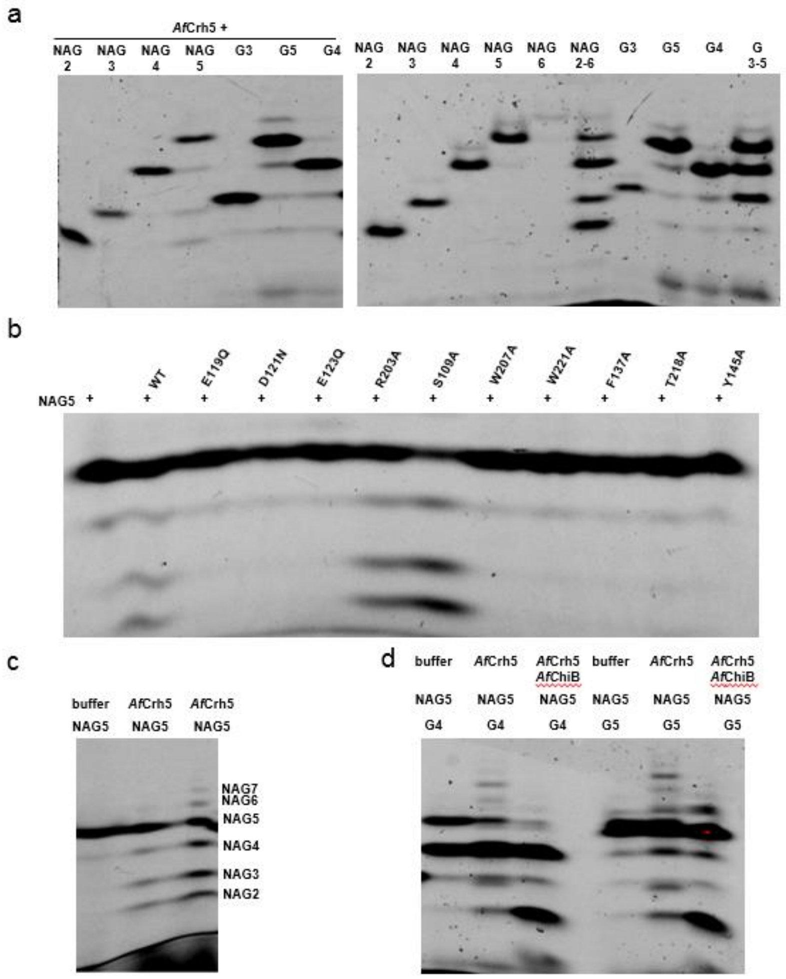

Supplementary Figure 7. Chitinase and transglycosylase activities were detected by FACE assay using 2.5 mM oligosaccharides incubated with or without 25 µg of proteins for 16 h at 37 °C in McIlvaine buffer pH 4.9. Reaction was stopped and labelled by 750 nmol ANTS as described in Methods**.** a) NAG2 to NAG5 and G3 to G5 were incubated with *Af*Crh5 WT protein. NAG2 to NAG6 and G3 to G5 sugar standards were loaded in parallel on FACE gel (right panel). b) NAG5 was incubated with WT and the indicated mutant enzymes of *Af*Crh5. c) NAG5 was incubated with or without WT *Af*Crh5 protein. Note the loading for lane 3 was double of lane 2. d) Transglycosylase activity using NAG5 as the donor, G4 or G5 as the acceptor. *Af*ChiB treatment was

applied at the indicated samples after the transglycosylation reaction. Source data are provided as a Source Data file.

Supplementary Table 1: PCR primers used in this paper. Restriction sites are denoted in bold.

Name	Sequence 5' to 3'	Details
P1	**gttaacTTAATTAA**CCTA**GGCCGGCC**CCTTG**cccggg**ACTCAT**GCGGCCGC**CTCCGCGATCGCCACC**GGCGCGCCgttaac**AGCT	*Making construct I*
P2	**gttaacGGCGCGCC**GGTGGCGATCGCGGAG**GCGGCCGC**ATGAGT**cccggg**CAAGG**GGCCGGCC**TAGG**TTAATTAAgttaac**GTAC	*Making construct I*
P3	aac**TTAATTAA**AACCTGTTTTCTACTGGGGACACCGC	*crh1 upstream*
P4	caagg**GGCCGGCC**GTTACTGTAAGGCTGTTGGCAGCAGATG	*Crh1 upstream*
P5	CTCAT**GCGGCCGC**ACCAACCAACATGTACTAGACTTTCTG	*crh1 downstream*
P6	aac**GGCGCGCC**GCTCCTGGTGCAGATGCGAAAGACC	*crh1 downstream*
P7	aac**TTAATTAA**ATCTGATTGGCTCAGAGCAGTTCATCG	*crh2 upstream*
P8	caagg**GGCCGGCC**GGTGGCTGATATTGTACAAGAGAATAG	*crh2 upstream*
P9	CTCAT**GCGGCCGC**GCGTGGGGTACTTGACACTTTCCAC	*crh2 downstream*
P10	aac**GGCGCGCC**AGAGCGGCCCTTCGCCTGGCTAGG	*crh2 downstream*
P11	aac**TTAATTAA**GATGATGAGAAGATGGATAAGATATGATTG	*crh3 upstream*
P12	caagg**GGCCGGCC**GATGGGGTTTTAGAAGGATCTCTCTCTAG	*crh3 upstream*
P13	CTCAT**GCGGCCGC**CCCTGCGTTGCAAAACGATCTCGTCC	*crh3 downstream*
P14	aac**GGCGCGCC**CGCGGCCGTCAGCTCCGCCATCGC	*crh3 downstream*
P15	aac**TTAATTAA**GAAGCATGGCGAGTTTCCCTTATAGG	*crh4 upstream*
P16	caagg**GGCCGGCC**GGTGAGAGGCTTCGGAGGTAATGTG	*crh4 upstream*
P17	CTCAT**GCGGCCGC**GCTGAGCTTACGAAGAAGCGATGCAC	*crh4 downstream*

P18	aac**GGCGCGCC**GGCTCCGAACTGACATTAGGGACCAAC	*crh4 downstream*
P19	aac**TTAATTAA**TTGAATGGAAGTTAGGATCTTTGATTCTC	*crh5 upstream*
P20	caagg**GGCCGGCC**GTCTGCGCTACGCAGGATAAAGGAGC	*crh5 upstream*
P21	CTCAT**GCGGCCGC**ACAAAATGACGATTTTGGTTTAATGCACG	*crh5 downstream*
P22	aac**GGCGCGCC**CAAGCGATTATCTGATCGATCAGGGAAG	*crh5 downstream*
P23	CCTGCTTATCTGCATCAAAT	*pyrG marker*
P24	TACGAATCAGGGTCCACCAG	*pyrG marker*
P25	GCGGGGATCTCATGCTGGAG	*Neo-d500*
P26	GGCGTAGTAGCTGTCCTCCT	*crh1-d-d*
P27	GTACAAGTGGCTTCCCGGGG	*crh2-d-d*
P28	AGAAGCCTGCAGAGCCTACA	*crh3-d-d*
P29	TGTCCTTCCACGAACATCAG	*crh4-d-d*
P30	ACAGTTCCGATCGACACAAG	*crh5-d-d*
P31	AAA**GGATCC**ATGATGCTGCCATTGCTGGCCGTTTC	*crh1 gene*
P32	ttt**GCGGCCGC**TTAGAACGCAAGTGCGATAGCCAGG	*crh1 gene*
P33	AAA**GGATCC**ATGGTGCGGATCGGCTCTTCACTTC	*crh2 gene*
P34	ttt**GCGGCCGC**TCACAGGGTGACCAGGGCAACAACG	*crh2 gene*
P35	AAA**AGATCT**ATGTCGCTCCTTTACCTTGTGGCCC	*crh3 gene*
P36	ttt**GCGGCCGC**TTAATTGACCGGCTGGTATCCCTTGTG	*crh3 gene*
P37	ATGAGATTGTCTCTCGTTGGTGTGGC	*crh4 gene*
P38	TCAAAAGATTGCGATGAGGCCGCCAAG	*crh4 gene*
P39	AAA**GGATCC**ATGTATTTCAAGTACACAGCAGCAGCC	*crh5 gene*
P40	ttt**GCGGCCGC**TTAGAATGCCAACACGGCAGCGACG	*crh5 gene*
P41	AAA**GGATCC**TGGTCAAAGTGCAATCCCCTTG	*Truncate of crh5 (22-275)*

P42	ttt**GCGGCCGC**TTA GCCCTGGGAGAGCTCGGGG	*Truncate of crh5 (22-275)*

Supplementary Reference:

1 Robert, X. & Gouet, P. Deciphering key features in protein structures with the new ENDscript server. *Nucleic Acids Res* **42**, W320-W324, (2014).
