# Supplementary material for: Mechanisms of redundancy and specificity of the Aspergillus fumigatus Crh transglycosylases
Source: Nat Commun. 2019 Apr 10;10:1669. doi: 10.1038/s41467-019-09674-0 (PMC6458159; doi:10.1038/s41467-019-09674-0)
Supplement: Supplementary file 3 — Reporting Summary [file 41467_2019_9674_MOESM3_ESM.pdf]

nature research

Corresponding author(s): Daan M. F. van Aalten

Last updated by author(s): Mar 14, 2019

Reporting Summary

Nature Research wishes to improve the reproducibility of the work that we publish. This form provides structure for consistency and transparency in reporting. For further information on Nature Research policies, see Authors & Referees and the Editorial Policy Checklist.

Statistics

For all statistical analyses, confirm that the following items are present in the figure legend, table legend, main text, or Methods section.

n/a	Confirmed	
☐	☒	The exact sample size (*n*) for each experimental group/condition, given as a discrete number and unit of measurement
☐	☒	A statement on whether measurements were taken from distinct samples or whether the same sample was measured repeatedly
☒	☐	The statistical test(s) used AND whether they are one- or two-sided<br>*Only common tests should be described solely by name; describe more complex techniques in the Methods section.*
☐	☒	A description of all covariates tested
☒	☐	A description of any assumptions or corrections, such as tests of normality and adjustment for multiple comparisons
☒	☐	A full description of the statistical parameters including central tendency (e.g. means) or other basic estimates (e.g. regression coefficient) AND variation (e.g. standard deviation) or associated estimates of uncertainty (e.g. confidence intervals)
☒	☐	For null hypothesis testing, the test statistic (e.g. *F*, *t*, *r*) with confidence intervals, effect sizes, degrees of freedom and *P* value noted<br>*Give P values as exact values whenever suitable.*
☒	☐	For Bayesian analysis, information on the choice of priors and Markov chain Monte Carlo settings
☒	☐	For hierarchical and complex designs, identification of the appropriate level for tests and full reporting of outcomes
☐	☒	Estimates of effect sizes (e.g. Cohen's *d*, Pearson's *r*), indicating how they were calculated

Our web collection on statistics for biologists contains articles on many of the points above.

Software and code

Policy information about availability of computer code

Data collection	Structural data were collected at the European Synchrotron Radiation Facility (ESRF) (Grenoble, France) at beamlines ID29 and ID30A-1. Enzymatic fluorescence was measured in a FluoStar Omega Reader (BMH Labtech). Fluorescent polysaccharides labeling photos were taken by fluorescence microscopy (Zeiss and Leica).
Data analysis	The diffraction data were processed with Imosflm. The apo structure was solved by molecular replacement using MOLREP with the modelled ScCrh1 structure as the search model, followed by WarpNtrace model building. The complexed structure was obtained by molecular replacement using the apo structure. In both cases, REFMAC was used for further refinement and iterated with model building using COOT. Images were produced by PyMol. Enzymatic data were analyzed by GraphPad Prism.

For manuscripts utilizing custom algorithms or software that are central to the research but not yet described in published literature, software must be made available to editors/reviewers. We strongly encourage code deposition in a community repository (e.g. GitHub). See the Nature Research guidelines for submitting code & software for further information.

Data

Policy information about availability of data

All manuscripts must include a data availability statement. This statement should provide the following information, where applicable:

- Accession codes, unique identifiers, or web links for publicly available datasets
- A list of figures that have associated raw data
- A description of any restrictions on data availability

The atomic coordinates and structure factors of AfCrh5 are available from Protein Data Bank with accession code 6IBU and 6IBW. Other data that support the findings of this study are available from the corresponding author upon reasonable request. A reporting summary for this article is available as a Supplementary Information file. The source data underlying Fig 2 and Supplementary Fig 2, 3 and 7 are provided as a Source Data file.

Field-specific reporting

Please select the one below that is the best fit for your research. If you are not sure, read the appropriate sections before making your selection.

☒ Life sciences ☐ Behavioural & social sciences ☐ Ecological, evolutionary & environmental sciences

For a reference copy of the document with all sections, see nature.com/documents/nr-reporting-summary-flat.pdf

Life sciences study design

All studies must disclose on these points even when the disclosure is negative.

Sample size	For in culture labeling experiment, the sample size is 3. For all enzymatic assays and crystallization trials, no simple size calculation was performed because a unique protein batch was used for each condition.
Data exclusions	No data were excluded from the analysis.
Replication	For in culture labeling experiment, three batches of conidia were used, no replication for each batch. For each enzymatic assay, three experimental replicates at three different days were carried out using exactly the same conditions (the same protein batch, the same substrates, the same buffer, etc). Reproducibility was successful as indicated by size of the errors bars.
Randomization	While capturing images from in culture labeling experiment, randomization was applied. This is not relevant for the enzymatic assays. There is no assignation of samples into experimental groups.
Blinding	Blinding was not required.

Reporting for specific materials, systems and methods

We require information from authors about some types of materials, experimental systems and methods used in many studies. Here, indicate whether each material, system or method listed is relevant to your study. If you are not sure if a list item applies to your research, read the appropriate section before selecting a response.

Materials & experimental systems

n/a	Involved in the study	
☒	☐	Antibodies
☒	☐	Eukaryotic cell lines
☒	☐	Palaeontology
☒	☐	Animals and other organisms
☒	☐	Human research participants
☒	☐	Clinical data

Methods

n/a	Involved in the study	
☒	☐	ChIP-seq
☒	☐	Flow cytometry
☒	☐	MRI-based neuroimaging
